# Ablative Techniques for the Management of Osseous Spine Metastases: A Narrative Review

**DOI:** 10.3390/jcm14186358

**Published:** 2025-09-09

**Authors:** Zach Pennington, Jonathan M. Morris, Aladine Elsamadicy, Sheng-Fu Larry Lo, Joseph H. Schwab, Daniel M. Sciubba

**Affiliations:** 1 Department of Neurologic Surgery, Mayo Clinic, Rochester, MN 55905, USA; 2 Department of Radiology, Mayo Clinic, Rochester, MN 55905, USA; 3 Department of Neurosurgery, Yale University School of Medicine, New Haven, CT 06510, USA; 4 Department of Neurosurgery, Zucker School of Medicine at Hofstra, Long Island Jewish Medical Center and North Shore University Hospital, Northwell Health, Manhasset, NY 11030, USA; 5 Department of Orthopaedics, Cedars-Sinai Medical Center, Los Angeles, CA 90048, USA

**Keywords:** cryoablation, spine metastases, epidural spinal cord compression, frailty, percutaneous technique, minimally invasive surgery, radiofrequency ablation (RFA), microwave ablation (MWA), laser interstitial thermal therapy (LITT)

## Abstract

With continued improvements in systemic cancer therapies, there has been an increase in the survivorship of patients with spinal metastases. However, many patients with spinal metastases are frail and may not be able to tolerate the morbidity of open surgery. For these patients, percutaneous ablation techniques offer a minimally invasive approach that can facilitate local tumor control and pain relief. Here we describe the currently employed modalities—radiofrequency ablation (RFA), microwave ablation (MWA), cryoablation, and laser interstitial thermal therapy (LITT)—summarize the clinical support for their use, and overview the relative risks and benefits for each. All these technologies offer to help improve local tumor control and improve oncologic pain associated with vertebral metastases, and they have become a staple of multidisciplinary spine metastasis care at many centers. As clinical experience with these technologies continues to grow, their use will likely become more widely adopted, and so understanding of their indications, risks, and benefits will become increasingly important to the practicing spine oncologist.

## 1. Introduction

In 2024, for the first time, there were more than 2 million new cancer diagnoses [1]. Of these, 40–70% may develop metastatic disease of the vertebral column [2], and upwards of 10% of these patients may require treatment for tumor-associated pain, deformity, or instability. With improvements in systemic therapy and radiation treatment paradigms, there have been massive improvements in expected survival [3], to the point that survival for many patients is discussed in terms of years rather than months. And for many pathologies, cancer has taken the form of a chronic disease, particularly those with oligometastatic disease involving the spine.

Despite these therapeutic improvements, frailty is common in the metastatic tumor population [4,5,6], and the high morbidity of open surgery may be unpalatable. Secondly, the timing of surgery can be difficult due to the need for radiation treatment and wound healing issues. Lastly, minimally invasive therapies for structural support in cases of impending fractures, large lytic lesions, and the treatment of painful pathologic fractures can prevent future morbidity and have been shown to significantly and efficiently reduce pain. In response to this, there have been advances over the past two decades focused on developing minimally invasive (MIS) interventional oncology methods for managing metastatic spine disease. These advances include cement augmentation (e.g., vertebroplasty or kyphoplasty), which has been used for years to help stabilize pathologic fractures in patients with osteoporosis and ablative techniques [7]. The latter are percutaneous techniques for tumor debulking, which can be paired with cement augmentation as an MIS strategy for patients with modest instability and oncologic pain. The present review focuses on the application of these ablative techniques to patients with metastatic spine disease, including technique, combination with cement augmentation, and patient selection.

## 2. Basis of Ablative Techniques and Patient Selection

**Figure 1:**
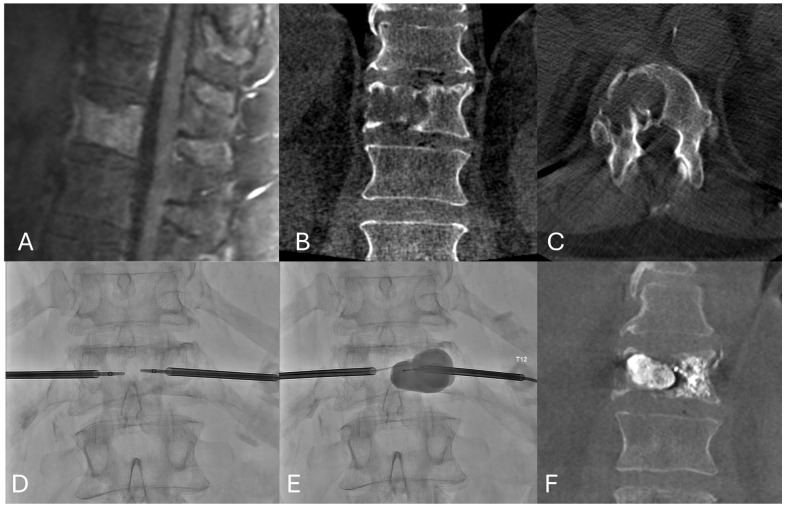
Palliative pain relief and structural support. A 72-year-old male with 3-year history of metastatic renal cell cancer presented with new onset of lower thoracic back pain. Radiation was performed, but due to the large lytic defect and ongoing pain limiting his activities of daily living, thermal ablation and kyphoplasty were performed. (**A**) Sagittal Post Gadolinium T1 weighted image demonstrating a pathologic fracture without epidural disease or retropulsion. (**B**,**C**) Coronal and Axial CT demonstrate more than 50% of the T12 vertebral body replaced by lytic metastasis. (**D**) AP fluoroscopic image demonstrating radiofrequency ablation using 20 mm bilateral Osteocool probes. (**E**) A 20 mm curved kyphoplasty balloon and 10 mm straight kyphoplasty balloon fill the lytic defect and are kept inflated during the left-sided PMMA cement filling to prevent extravasation, and finally deflated, and the lytic cavity is filled. (**F**) Coronal CT demonstrating results of post-RF ablation kyphoplasty.

**Figure 2:**
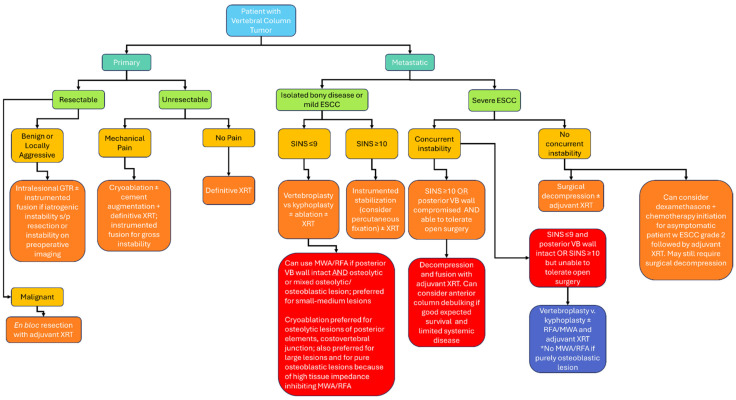
Proposed algorithm for the incorporation of ablative technologies into the management of patients with bony spine tumors. Key: ESCC—epidural spinal cord compression. GTR—gross total resection. LITT—laser interstitial thermal therapy. MWA—microwave ablation. RFA—radiofrequency ablation. SINS—spinal instability neoplastic score. VB—vertebral body. XRT—radiotherapy.

The primary ablative technologies (Table 1) include cryoablation, radiofrequency ablation (RFA), laser interstitial thermal ablation (LITT), and microwave ablation (MWA) [7]. All use percutaneously placed probes that are inserted into the target tissue through 10–13 gauge trocars under image guidance, through which thermal damage, either by heat or cold, causes necrosis of tumor cells within the ablation zone. The goals of percutaneous ablative therapy are pain relief, structural support, preventing neurologic decline, and, in some cases, local tumor control (Figure 1) [8]. Consequently, it has become a viable option for patients (1) with pain refractory to or inadequately treated by radiotherapy, (2) with contraindications to radiotherapy, (3) in concert with radiation therapy in patients with risk of fracture or fracture worsening [9,10,11], (4) with inadequate local control under radiotherapy, (5) previous non-durable response to radiation at another index spinal level, (6) primary treatment for local recurrence despite previous surgical and radiation therapies [12,13] (7) local tumor control in oligometastatic disease for certain pathologies [14,15,16,17,18], (8) reduced hormonal therapies in oligometastatic prostate cancer [19], (9) reduced physiologic symptoms in biologically active metastasis [13,20] such as those secreting catecholamines [8]. The exact criteria determining patient candidacy vary by center, but in general, patients must have one or more of the following but do not need all: (1) metastatic disease to the vertebral column that is progressing on systemic therapy despite effective disease control elsewhere, (2) oligometastatic disease (defined as <3 bone metastases—each ≤3 cm—and no or minimal visceral disease burden), (3) pain localized to the sites of metastatic disease with at least moderate pain (>4/10 on visual analog scale), (4) low-grade epidural disease burden (Bilsky 0–1 c) and minimal paraspinal spread, and (5) large lytic defect with impending fracture undergoing radiation [13,20]. Patients should generally only have modest instability at the treated level with a minimum of 5 mm between the edge of the target ablation zone and the neural elements if attempting local control. However, for palliative pain relief in patients with limited life expectancy (<6 months), it is reasonable to consider treatment of lesions with <5 mm between the tumor and ablation zone, especially if being used in combination with radiotherapy. Some authors recommend ablative techniques only for stable lesions, defined as those with a spinal instability neoplastic score (SINS) less than 7 [8]. However, this is not a consensus opinion, and for patients who are not surgical candidates, ablation can be performed for unstable lesions with significant associated pain, such as pathologic odontoid fractures, so as to maximize the patient’s quality of life. We provide a suggested algorithm for the incorporation of ablative technologies in the management of patients with bony spine tumors (Figure 2).

## 3. Cryoablation

**Figure 3:**
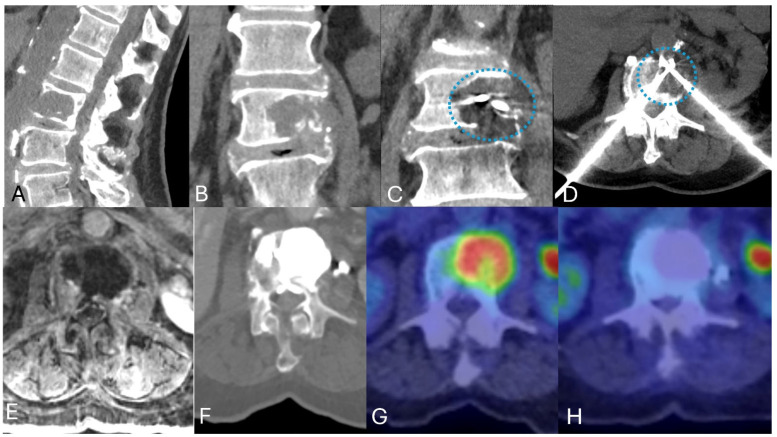
Cryoablation for curative intent. An 82-year-old female smoker with a history of previously radiated L2 metastasis presented with new onset lumbar pain 1 year after radiation. Studies demonstrated worsening of her L2 metastasis and a painful pathologic fracture. (**A**,**B**) Sagittal and coronal CT demonstrate a pathologic fracture through a lytic metastasis. (**C**,**D**) Coronal and Axial MIP images demonstrate two variable cryoablation probes crossing in the lesion and associated ice ball (dashed circle) at 8 min. (**E**) Post Gadolinium T1WI with fat saturation demonstrates the thermal margin 3 months after the procedure. (**F**) Axial CT demonstrates complete filling of the large lytic defect with PMMA cement, providing structural support. (**G**,**H**) Axial FDG PET/CT prior to treatment and 1 year after ablation.

**Figure 4:**
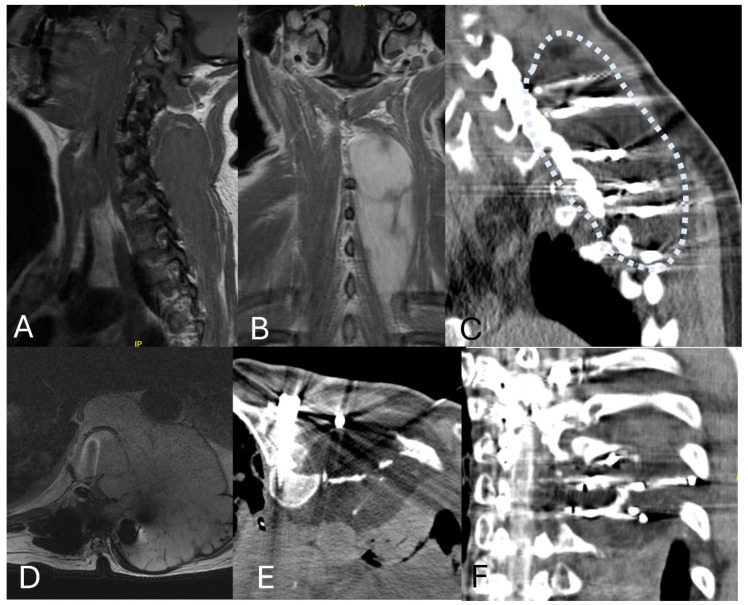
Cryoablation of large tumors: (**A**,**B**) Sagittal T1WI and Coronal Post Gadolinium T1WI demonstrate a 13 cm infiltrating paraspinal desmoid tumor in a 42-year-old female. (**C**) A total of 10 cryoablation probes were used, starting 1.5 cm from the superior margin and stacking probes vertically 2 cm apart. Sagittal CT demonstrates the ice ball with a 5 mm thermal margin around the lesion. (**D**) Axial T2WI demonstrates a large recurrent chondrosarcoma growing into the epidural space despite previous separation surgery and radiation. (**E**,**F**) After surgical removal of the epidural component through a smaller incision, the remaining tumor was cryoablated twice for 8 min. The patient lived 2.5 more years and died due to pulmonary metastasis burden.

Cryoablation—also known as cryotherapy or cryosurgery—employs freezing to destroy target tissues, and modern cryosurgery devices have been applied to bony lesions since the 1960s. However, real-time image guidance to monitor tissue lesioning has only become possible in the last 10–15 years. The first published report of cryoablation for vertebral metastasis was published by Masala and colleagues [21], who described the treatment of an L2 cholangiocarcinoma metastasis with associated pathologic compression fracture causing 40% vertebral height loss. The patient subsequently underwent vertebroplasty with polymethylmethacrylate (PMMA) cement. At one author’s institution, cryoablation of the spine for primary and metastatic disease has been performed for two decades, starting in 2005, and was the dominant methodology for the decade preceding modern-day spine-specific ablation devices (Figure 3 and Figure 4) [13].

Cryoablation devices work by circulating cooling fluid or, more commonly, exploiting the Joule–Thomson (J–T) effect, where cooling is caused by rapid expansion of high-pressure argon gas through a narrow opening within the cryosurgical probe [22,23], resulting in freezing the tissue. The freezing interface then spreads radially from the probe, creating an “ice ball” in which tissue temperatures range from the cryogenic probe temperature to the phase transformation temperature—the temperature at which tissue freezes. The tissue is then held at this temperature for several minutes, after which it is allowed to thaw, which occurs due to the passive influx of heat from the circulation and metabolic activity in the surrounding tissue or actively using high-pressure helium gas. Cryoablation results in cell killing through a combination of (1) membrane protein dysfunction and resultant ionic gradient disruption, (2) mechanical damage of cytoskeletal elements, (3) protein denaturation, and (4) occlusion of small blood vessels with resultant ischemia. With rapid freezing, there is also the formation of intracellular ice, which correlates with higher levels of cell death even at higher cryoablative temperatures (e.g., 80% death at −10 °C versus −40 °C with slower cooling). Double or triple freeze–thaw cycles are also commonly used as they have shown increased cell damage relative to single freeze–thaw cycles, likely because of increased cell membrane damage.

**Figure 5:**
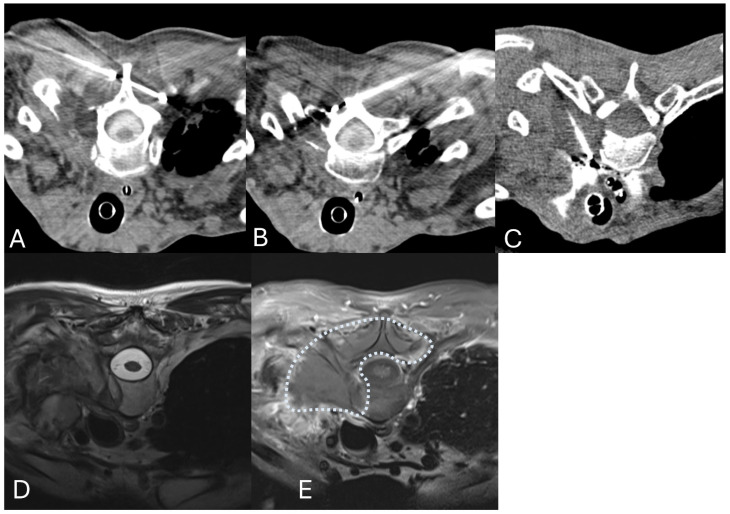
Cryoablation allows shapable thermal margins: (**A**–**C**) Variable-sized cryoablation probes placed under CT guidance over the lamina of T1 and adjacent to the spine at T1/2 on the right with thermal ice ball (hypointense region around the probes) at 8 min. (**D**,**E**) Axial FSE T2WI and Axial fat-saturated T1WI after gadolinium allow visualization of the thermal margin 6 months after treatment (dashed line).

Patients are typically placed under general anesthesia, although simpler, smaller number of probe cases can be performed under conscious sedation. In cases where the ice will be immediately adjacent to the thecal sac or critical nerve, total IV anesthesia (TIVA) is employed to also perform neural monitoring with motor evoked potentials (MEP) and, in some cases, sensory evoked potential (SEP) to improve safety [24]. In selecting a probe trajectory and region for ablation, it is important to identify the adjacent neural elements. The goal for local control is to select an ablation zone that extends 3 mm beyond the margin of the tumor, or in combination or palliative cases, abuts the edge of the tumor but spares the neural tissue at risk. Typically, multiple probes are arranged to shape the ice to the desired metastasis, for example, cryoprobes overlying the lamina, crossing probes in the vertebral body, or arranged in series and parallel for large tumors 1.5 to 2 cm apart [25]. Cryoablation has been traditionally performed within interventional CT suites where pilot holes are created using bone access needles or hand drills under CT guidance, and then the probes are placed within the pilot holes (Figure 5). The ice ball can be monitored by CT scanning every two minutes, and visualizing the low-density ice ball formation. More recently, as MRI-compatible probes have been created and MRI thermography has become available, monitoring during the procedure can be performed with thermometry or impedancemetry (tissue impedance rises in frozen tissue) [22]. However, MR-guided thermometry using T1-weighted sequences has been employed at larger centers and takes advantage of the different relaxation times of protons in frozen and unfrozen tissue. MR monitoring is more costly and can be more tedious than CT-based monitoring, which remains to be the favored monitoring modality at many locations. As a treatment modality, cryoablation is costly, requires gas infrastructure, and requires more operator training which may restrict its use to tertiary care centers. Yet it has the advantage over other ablative procedures of treating larger lesions and more vascular lesions, ability to shape the thermal ablation zone, has a history of proven durable local control than newer modalities, and partial analgesia can occur rapidly as cooling leads to decreased nociceptor activity [21,23,26]. Additionally, it has the advantage over focused radiation of leading to rapid pain improvement and local control independent of underlying tumor radiosensitivity [27]. Cryoablation can also be paired with cement augmentation, reducing the pathologic fracture risk as well as providing stabilization for vertebrae that have already fractured, which is often seen following radiotherapy [11,28].

### Published Outcomes

In a recent systematic review of eight studies (148 patients and 187 lesions), Sagoo and colleagues [29] examined pain improvement in patients treated with cryoablation for spinal metastases of all bone quality (lytic, blastic, and mixed) and regions of the spine. Lesions of both the vertebral body and posterior elements were treated, and 53% of the patients were treated under conscious sedation, eliminating the risk of general anesthesia that is of concern in frail patients. Patients experienced an average 5-point improvement in pain scores at 1-month follow-up and 4.6 points at last follow-up. Local tumor control was achieved in 58% at last follow-up, and only 8% of the patients suffered a complication (three major). Thirty-five percent of patients were able to undergo cryoablation paired with cement augmentation as described by Masal and colleagues [21].

Subsequently, Cazzato et al. [27] described using a commercially available cryoablation system with a 10 min double freeze–thaw cycle in 74 patients (105 lesions), a pooled cohort including many patients previously reported in the Sagoo et al. [29] meta-analysis. For palliative attempts, the ice ball margins were planned at the metastasis/normal bone interface and in curative attempts the margins were planned 5–10 mm beyond the metastasis borders based on intra-procedural CT. All the cases used a safety margin of 1 cm around critical structures and employed thermometry with or without concomitant neuromonitoring to monitor ice ball size and adjacent neural tissue irritation. Adjuvant PMMA cementoplasty was used for those deemed at high risk of pathologic fracture and curative cryoablation was attempted in 28%. Patient with pre-procedural pain had an average 2.5-point improvement at 24 h, 4.3-points at 1-month, and 4.4-points at last follow-up. Complete pain relief was achieved in 53%, local control in 82% of those treated with curative intent, and only 9% of treatments resulted in complications, the majority of which were neural injuries that recovered spontaneously or improved significantly with local steroid injections. While this experience remains small, it suggests cryoablation may be an effective option for patients with spinal metastases. Additionally, work from the same group comparing RFA and cryoablation suggested similar complication profiles, but found cryoablation to be associated with lower peri-procedural pain [26]. They also recommend cryoablation over other ablative techniques, specifically RFA, where lesions are large (≥3 cm) or non-lytic.

## 4. Radiofrequency Ablation (RFA) and Microwave Ablation (MWA)

### 4.1. RFA

**Figure 6:**
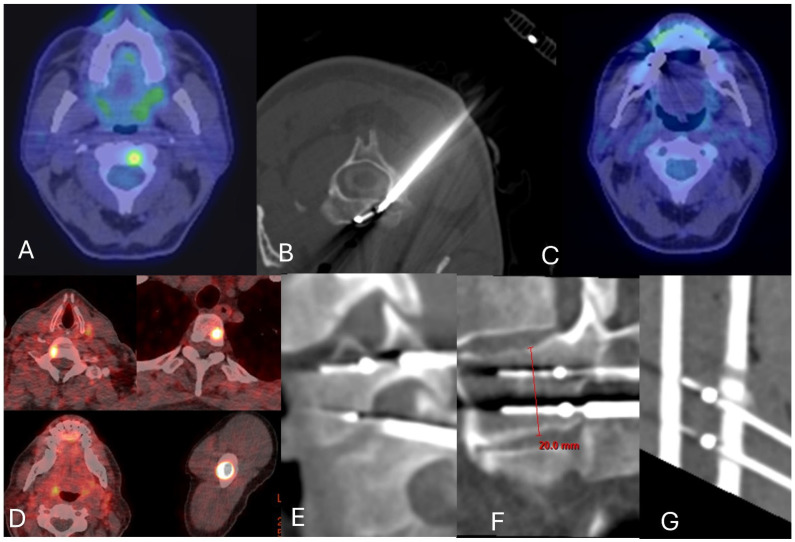
Radiofrequency ablation with curative intent. A 76-year-old male with 15-year history of metastatic prostate cancer and rising PSA who recently underwent treatment with lutetium-177 Dotatate. (**A**) Axial PMSA PET demonstrated a solitary metastasis at C2. (**B**) Curved Star Tumor ablation system was placed transpedicularly, and lesion was ablated for two cycles. (**C**) Follow-up PMSA PET at 1 year demonstrated no recurrence. (**D**) 68 Ga Dotatate PET study demonstrates oligometastatic disease involving T1, T3, and the left humerus in a 35-year-old male with SDHB-mutated paraganglioma. (**E**) Angulated vertically stacked 10 mm Osteocool probes placed transpedicularly and using an inferior pedicle approach into T1. (**F**) Vertically stacked 15 mm Osteocool probes within the T3 lesion. (**G**) Vertically stacked 7 mm Medtronic Osteocool probes. Follow-up out to 3 years shows no recurrence.

RFA was the first thermal ablation technology to be purposely made for the palliative ablation of spine metastasis in 2004, with the approval of the D-fine star ablation curved probe (Figure 6). Later two additional devices were FDA approved for palliative thermal ablation of the spine: Osteocool (Medtronic, Dublin, Ireland) in 2016 and Optablate (Stryker, Kalamazoo, MI, USA) in 2022, making RFA the most commonly employed modality for the treatment of painful spine metastases [30]. Patients are placed prone on a fluoroscopic or CT table, and the procedure can be performed under general anesthesia or conscious sedation with local anesthetic [31,32]. Fluoroscopy or intraoperative navigation significantly aids in trajectory approaches when compared to CT, which is typically reserved for cryoablation. The planned ablation trajectory is infiltrated with 1–2% lidocaine at the skin and 0.25% bupivacaine on the periosteum for local analgesia, and a 10–13-gauge bone access needle is placed under image guidance to create a pilot hole. The needle is then retracted to the pedicle vertebral body junction in most cases, and the RFA probes are placed coaxially through them, positioned in the center of the target lesion for local control or 8–10 mm apart at the tip for palliative tumor ablation preceding cement augmentation [33]. Bipedicular approaches are commonly used, as the ablative zones of the two probes can create a larger, confluent ablation zone [8,32]. Grounding pads may need to be placed elsewhere on the patient with older RF systems to complete the electric circuit and to improve safety, but most modern-day bipolar systems do not require them. A thermocouple can be simultaneously placed in the epidural or perineural space to allow for monitoring of spinal canal temperature [8]; however, many modern RFA systems now come with integrated thermocouples [34]. Using a threshold of 45 °C can help reduce the risk of spinal cord injury, and probes with integrated thermocouple may have automatic shut-off features that stop energy delivery when the proximal thermocouple reaches 50 °C [32]. Additionally choosing the right size probe for the vertebrae, goals of treatment, and size of lesion is crucial to mitigate complications such as neural injury.

Both monopolar and bipolar RF systems exist; however, bipolar systems have an increased degree of safety, as current flows only between the two tips and they have lower temperatures near the electrode [34], both of which can reduce the risk of injury to nearby neural structures [30]. Articulating RF probes are now also commercially available and have the advantage of allowing treatment of lesions centrally located along the posterior vertebral body, which are difficult or impossible to access with conventional probes [8]. Unlike cryoablation, RFA is generally reserved for lytic or mixed osteolytic/osteoblastic lesions; sclerotic lesions are seldom amenable to this modality unless they have previously undergone radiation therapy due to their high impedance [8]. Tumor killing in RF occurs when 450–500 Hz electrical current is passed through the tissue, heating it and causing coagulative necrosis [35]. Target temperatures at the RF probe tip can be as high as 100 °C [33], though lower target temperatures of 60–75 °C may be preferable if the cortical bone is compromised, as the high impedance of cortical bone allows it to function as a thermal barrier [36]. Heating times range from 3 to 7 min [33].

### 4.2. MWA

MWA similarly involves placing the probe transpedicularly into the vertebral body after a pilot hole is made under fluoroscopy or CT-guidance [37] and can be performed under general anesthesia or moderate sedation depending upon patient frailty and ability to comply with lying still in the prone procedure. An access cannula guides a hand drill to the pedicle edge, which is then advanced to just beyond the edge of the tumor/ablation target. The ablation antenna is then passed coaxially to approximately 2 mm beyond the target lesion, and a thermocouple is placed separately in the epidural space or juxtaforaminally to monitor temperature changes during ablation. A curved access trocar and flexible antenna can be used for lesions of the posterior body [37]. MWA uses higher frequency energy than RFA, employing 915 MHz or 2.45 GHz microwave generators, which cause heating through the induced oscillation of polar molecules in tissue adjacent to the antenna [38,39]. This is as compared to RF ablation, where heating occurs from the passage of electrical current through an ionic tissue medium [39]. Consequently, MWA can conduct energy even through low electrical conductivity/high impedance tissues (e.g., bone), which allows for faster ablation times [39]. MWA can also take advantage of thermal synergy, facilitating the use of multiple probes simultaneously. Some argue this may lower the risk of neural injury [37], yet others suggest RFA is safer and causes less damage to surrounding normal tissue [40]. RFA remains the most common thermal ablation device in the spine to date due to several factors that include safety.

### 4.3. Published Outcomes

In a 2018 systematic review, Cazzato et al. [30] summarized the results of 8 studies on RFA comprising 261 patients and over 360 unique lesions. Mean ablation times ranged from 6 to 9.75 min, and temperatures ranged from 50 to 100 °C; most patients also received adjuvant cement augmentation, generally as part of a single procedure. Overall pain relief was good, with 30–75% of preoperative pain being relieved. In all studies there was a significant decrease in pain scores from the preoperative state. Two studies also compared pain outcomes following RFA with cement augmentation to RFA alone and found greater improvement with the addition of cement augmentation. Complications occurred in 16% of cases and were generally mild. Last, they noted local control in 77–92% at 3 months and 67% at 2 years; however, they recommended an attempt at local cure be reserved for lesions <2 cm in size without significant extension beyond the vertebral body. A subsequent series of 60 patients (75 lesions) published by Abdelgawaad et al. [41] noted significant pain improvement by post-procedure day 3 (2.7 ± 1.9 vs. 7.2 ± 2.3) that persisted at 6-month follow-up (3 ± 2.1); there were no neurological complications in their series. Similarly, positive results have also been reported by Senol et al. [42], who noted good pain relief and improvement in functional status at 6-month follow-up with only 2 transient motor deficits postoperatively.

A recent series by Motaghi et al. examined pain outcomes in 28 patients treated with MWA for spinal metastases [37]. Procedural time was rapid (median 4 min 13 s) with a median ablation temperature of 80 °C and no procedural complications. Average VAS pain scores decreased from 8 to 1 post-procedurally and remained at 1 through 6-month follow-up. All the patients had a reduction in the PET activity, consistent with reduction in tumor burden, and 16 had no PET activity during follow-up. These results concorded with those of an earlier systematic review by Sagoo and colleagues, which encompassed eight studies with 156 aggregate patients treated for 196 lesions [38]. A combination of open and percutaneous MWA procedures was included, with a mean power of 30–62.9 W and treatment with 3.8–4.8 ablation cycles. Good pain relief was reported with a 33–70% pain reduction by the last clinical follow-up, and only 6.5% of the cases experienced complications. Local tumor control was also high, reported at 88–96% at 6-month follow-up, and one series reported 79% control at 23-month follow-up. Yao et al. [40] subsequently compared the outcomes of MWA and RFA in a systematic review of 16 studies encompassing 630 patients (393 treated with MWA). Both treatment modalities led to significant pain improvement (93–100% vs. 54–100%), and local tumor control was >80% in both cases; however, complications were more common following MWA (27.4% vs. 10.9%). The authors noted MWA may potentially offer superior local tumor control due to the susceptibility of RFA to high impedance in bony tissue and the potential carbonization around the RFA tip as tissues are heated.

The OsteoCool Tumor Ablation Post-Market Study (OpuS One Study) also provided evidence for the effectiveness and safety of RF for painful spine metastases [43,44]. In this multicenter trial, 100 patients (87 thoracolumbar metastases; 13 sacropelvic metastases) underwent RF ablation of 134 total sites at one of 14 international centers; in 97% of the cases, this was followed by PMMA augmentation. Patients noted significant pain relief by postoperative day 3 (5.6 ± 2.7 vs. 8.2 ± 1.7), which persisted to 6-month follow-up (3.5 ± 3.2) in the initial study [43]. Patient similarly reported significant improvements in quality of life, and none of the procedures suffered procedure-related complications. In the expanded follow-up cohort of 205 patients with 12-month follow-up [44], nearly 60% of the patients reached the minimum clinically important difference (MCID) for pain relief by 3 days postoperatively, and 74% had a complete (27%) or partial pain response (47%) by 12 months. Additionally, there were significant improvements in quality of life at all follow-up time points, starting at postoperative day 3, with a decrease in opioid usage in 34–51% of patients at each follow-up. Ninety-seven percent of patients underwent post-ablation cement augmentation, and only 2.9% had procedure-related complications (1.5% with serious adverse events).

## 5. Laser Interstitial Thermal Therapy (LITT)

LITT is the newest percutaneous ablative technique and, like the other ablative techniques, can be paired with stereotactic radiosurgery to treat epidural disease [45]. First employed for brain tumors in 1983 [46], its use has become more widespread secondary to improved MRI thermometry, the creation of MRI-compatible probes, and the development of MRI thermometry software capable of measuring the cumulative damage of serial LITT treatments.

LITT systems employ class IV solid-state diode lasers with 2–40 W output power and wavelengths in the near-infrared spectrum [47]. They employ total internal reflection to carry energy along flexible optical fibers (≈10 m in length) to a 3–25 mm diffusing tip placed within the target tissue. The diffusing tip then distributes the laser energy radially, heating the surrounding tissue. Laser power and exposure time determine lesion size, and MR thermometry measures local tissue temperatures through changes in the water proton resonance frequency that occur with heating. Tissue damage occurs above 42.5 °C, though general cutoff targets of <50 °C are used on critical structures. Probes are placed stereotactically, as has been described by Vega and colleagues [45]. Because of the need to keep the patient still within the MR during ablation, the procedure is performed prone with the patient under general anesthesia [45]. During treatment periods, breath holds up to 120 s may be required, and close monitoring of O_2_ saturation is necessary; it is recommended to pause treatment and resume ventilation if the SpO_2_ falls below 94% [48]. Consequently, while LITT minimizes procedural morbidity relative to open or even MIS surgery, patients are still subjected to the physiological stresses of anesthesia. Nevertheless, the overall physical insult is lower, and so it may be a good option for frail patients [45]. Available clinical experiences are small; however, they have described a role for LITT that is similar to the role of open decompression under the separation surgery paradigm [49]. Under this paradigm, LITT [or open surgery] acts as the neoadjuvant therapy for stereotactic radiosurgery, reducing the epidural disease so as to create a safe boundary between tumor and spinal cord and thereby facilitate delivery of a therapeutic radiation dose [45].

### Published Outcomes

Most of the work on LITT for metastatic disease has come from MD Anderson Cancer Center [45,48,50,51,52,53,54]. Tatsui et al. [50] provided the first description, which used the Visualase^®^ (Medtronic, Minneapolis, MN, USA) system—a 980 nm diode connected to a 30 W power source (the group used a 15 W source in subsequent descriptions). Fibers were placed under fluoroscopic and stereotactic guidance using oblique transpedicular and transforaminal trajectories for ventrolateral epidural disease and a contralateral translaminar trajectory for dorsal/dorsolateral disease. All trajectories were kept a minimum of 5–6 mm from the thecal sac, and the authors employed temperature cutoffs of 50 °C at the dura-tumor interface and 90 °C within tissue adjacent to the laser fiber. Among their eleven treated patients, they reported a median tumor ablation of 36% using a median of 2 fibers. Median hospital stay was 2 days, and only one patient suffered transient neurological symptoms (paresthesias). Patients endorsed a significant improvement in pain (≈2 points at 30-day follow-up and 3.3 points at 60-day follow-up). In the follow-up description of 19 patients (28 treated levels) [48], pain improvement was again good at 1- (2.56 ± 0.71 vs. 4.72 ± 0.67) and 3-month follow-up (3.25 ± 0.75 vs. 4.72 ± 0.67), and there was a significant improvement in quality of life at 3-month follow-up (EQ-5D 0.83 ± 0.06 vs. 0.67 ± 0.07). Follow-up imaging also showed significant radiographic decompression at 2 months with a 22% median reduction in epidural tumor thickness. Based upon these preliminary experiences, they made several recommendations [45]. Under their paradigm, they employed an average of three burn cycles to ensure adequate ablation, withdrawing the fiber 2–3 mm between each treatment. They recommended LITT be reserved for those patients with vertebral body lesions contained by the posterior longitudinal ligament and recommended against treatment of isolated high-grade epidural disease owing to the limited ability to achieve complete ablation. They additionally recommended surgery for patients with epidural spinal cord compression (ESCC) causing neurologic deficits, as the ablation-related decompression takes 2–3 weeks to occur.

In their most recent description [51], encompassing 129 patients treated at 144 segments, the group reported local disease control in 80% of cases at 1 year. They found that poorer local disease control was predicted by the presence of preoperative paraspinal or foraminal disease, and a post-LITT epidural disease burden grade of Bilsky 2 or 3. Patients were able to be successfully radiated at a median of 5 days following LITT with one of the following schemas: 18–24 Gy/single fraction, 24–27 Gy/three fractions, or 30–40 Gy/five fractions (larger targets or previously irradiated lesions). Median hospitalization was 2 days, and complications occurred in only 12% of cases. The average patient had a median improvement in epidural disease burden by 1 Bilsky grade. However, in their earlier experience of 110 patients (120 lesions) [52], the authors noted neurological complications in 7.5% of cases, with an increased risk of complications with the treatment of cervical or lumbar lesions, likely due to the eloquence of nerve roots at these levels. Consequently, the authors recommended limiting LITT to the treatment of lesions of the T2–12 vertebral bodies contained by the PLL, who are to undergo adjuvant radiotherapy shortly following the LITT procedure. For these patients, LITT with adjuvant radiotherapy has been found to offer similar progression-free survival to open decompression but with a lower complication rate and shorter times to both initiation of radiotherapy and resumption of systemic therapy [55].

## 6. Pairing with Cementoplasty/Percutaneous Stabilization

Many studies pair ablation procedures with cementoplasty/cement augmentation, as ablation alone does not increase the stability of tumor-involved vertebrae [40]. In fact, some have suggested that RFA alone may increase spinal instability and the risk of subsequent pathologic fracture [56]. The combination of ablation and cement augmentation—first described by Schaefer and colleagues [57]—has shown improved pain relief beyond that offered by ablation alone. In the OPuS One study [44], 206 subjects underwent RFA for painful spine or pelvic metastases; 97% underwent cement augmentation as well. Patients on the whole had excellent pain relief, with 59.8% reaching the MCID for pain improvement by postoperative day 3, and 74% having complete or partial pain response at 12 months. Another small series by Nakatsuka et al. found patients treated with RFA and cement augmentation had a more than two-point greater improvement in their pain [31]. Proschek et al. similarly noted a trend toward improved pain relief in patients treated with cement augmentation in addition to RFA versus RFA alone, though the study was underpowered [58]. More recently, a retrospective cohort study [59] comparing 26 patients treated with RFA alone to 40 treated with RFA and vertebroplasty demonstrated significantly lower pain scores in those treated with vertebroplasty at 6-month follow-up (2.31 ± 1.42 vs. 4.41 ± 1.08), though pain scores were grossly similar for the first month of follow-up. Interestingly, a contemporaneous study of 64 patients published by Jain and colleagues [60] suggested the addition of RFA to cement augmentation may not improve pain control above that offered by cement augmentation, raising the question of the additive benefit of ablative procedures to cement augmentation alone. The benefit appears in the form of improved local tumor control [61], in creating a warmer environment and cavity in the target bone for receipt of the PMMA during vertebral augmentation [33,59], in reducing the number of circulating tumor cells displaced into the circulation by the cement, and by debulking assist in prevention of displacing soft tissue tumor through osseous defects into the foramen and epidural space. This may reduce the rate of cement extravasation, a feared complication of the cement augmentation of vertebral column metastases. Results from Lv et al. supported both advantages, noting significantly improved local tumor control (local recurrence in 11.4% vs. 30.8%; *p* = 0.036) and lower rates of cement extravasation (6.4% vs. 18.1%; *p* = 0.033) [62].

## 7. Limitations

There are several limitations to the present review. Foremost is that the majority of data on ablation technologies is derived from small- to medium-sized retrospective cohort studies. In the case of LITT, the data are almost wholly derived from the experiences of a single center. Accordingly, the overall quality of evidence is low, with the best data supporting the benefits of RFA. To the same end, much of the available data for ablation technologies is derived from the experiences of high-volume tumor centers. While outcomes in other disciplines have suggested care is better at these centers, it also limits the generalizability of the present results. Third, though we propose a framework for the incorporation of ablative technologies in the management of patients with bony spine tumors, there is a paucity of literature comparing outcomes between RFA, MWA, LITT, and cryoablation. In part, this is because of the novelty of LITT and the high expenses associated with cryoablation and LITT technologies relative to MWA/RFA. Additionally, at many centers, only one of these technologies is employed—that which the provider feels most comfortable with—and so it is impossible to eliminate all confounders when comparing outcomes between groups.

## 8. Conclusions

Given the convergence of decreasing oncological mortality over the last 20 years, the increase in incidence of patients diagnosed with cancer, the increased number of patients living with cancer, the improvement in early detection and treatment modalities, and the increased survival of those with metastatic disease, the number of patients with spinal metastases continues to increase. However, as frailty is common within this population, the number of patients requiring intervention for painful spinal metastases who are too frail for surgical intervention has also risen. Percutaneous ablation techniques, especially when paired with percutaneous cement augmentation, offer a minimally invasive option for durable and quick pain relief, structural vertebral support, and improved local control in this population. Given the expansion of device choices, advances in technologies, and monitoring capabilities of the thermal margin, they offer an alternative for achieving local tumor control and pain relief in patients with oligometastatic disease and pain attributable to one or two lesions without overt spinal cord compression. Experiences with LITT are limited, but RFA/MWA appear effective for smaller lesions (<3 cm), and cryoablation may be a reasonable option for sclerotic lesions, larger lesions, and hypervascular lesions. All the techniques are likely part of the armamentarium of a multidisciplinary spine tumor team, and comfort with these technologies will likely continue to increase with the training of additional staff, larger number of centers offering interventional oncology solutions, needs of our patient populations, and continued publication of positive results.

## Figures and Tables

**Table 1:** Summary of ablative technologies used for the management of spinal metastases.

Technique	Details	Target Patients and Limitations	Outcomes
MWA	Probes placed transpedicularly under CT- or fluoroscopic guidance. Epidural or perineural thermocouple to monitor canal temperature. Kill tumor with 915 MHz–2.45 GHz microwaves, causing coagulative necrosis. Can use multiple probes simultaneously—thermal synergy that can allow for faster ablation.	*Patients* Prefer VB lesions with intact cortical bone. *Advantages* Can be performed under moderate sedation [vs. general anesthesia]. Cheaper than LITT or cryoablation. *Disadvantages* May cause more damage to the surrounding tissue than RFA. Cannot monitor killing on CT.	Local control: 88–96% at 6 mo; 79% at 2 yr. Pain relief: 33–70% of patients with complete relief; some relief in 54–100%. Complications: 6.5–27.4%.
RFA	Most common modality for treatment of painful spine metastases. Probes placed transpedicularly under CT- or fluoroscopic guidance. Kill tumor with 450–500 Hz current, causing coagulative necrosis. Optional thermocouple placement in epidural/perineural space to monitor canal temperature.	*Patients* Prefer VB lesions with intact cortical bone. Local cure for lesions <2 cm in size. *Advantages* Strongest evidentiary support for pain palliation. Cheaper than LITT or cryoablation. *Disadvantages* Heating above 45 °C can risk spinal cord injury. Cannot monitor killing on CT.	Local control: 77–92% at 3 months; 67% at 2 years. Pain relief: 30–75% with complete relief; 93–100% with some pain relief; average ≈ 50% decrease in pain post-procedure. Complications: 10.9–16%.
LITT	Use class IV solid-state near-infrared diode lasers (2–40 W output) with 3–25 mm diffusing tip fibers. Use MR thermometry to monitor tissue killing.	*Patients* Able to tolerate general anesthesia with breath holds up to 120 s. T2–12 VB lesions contained by the PLL; has been used for epidural disease. *Advantages* Can monitor real-time tumor killing on MR. *Disadvantages* Must be performed under general anesthesia with breath holding during ablation. Expensive.	Local control: 22% reduction in epidural tumor burden; local control in 80% at 1 year. Complications: neurological complications in 7.5%.
Cryoablation	Oldest technique Real-time monitoring with CT or MRI Freeze ablation tissue by circulating cooling fluid or expansion of high-pressure argon case in cryosurgical probe; kills through microvascular thrombosis. Rapid freezing allows for greater cell death with less extreme temperatures; double or triple freeze–thaw cycles are used to increase cell killing. Neuromonitoring with MEP ± SEP when the lesion is adjacent to the cord.	*Patients* Large tumor (uses multiple cryoablation probes). Vascular lesions. A 3 mm margin between tumor and neural element if local control is sought. *Advantages* Concomitant pain control. Can visualize an ice ball on CT. Tumor control independent of radiosensitivity. *Disadvantages* Facility must have gas infrastructure and requires greater operator training than MWA or RFA.	Local control in 58%. Pain relief: 53% with complete pain relief. Complications: 8–9%.

Key: CT—computed tomography; LITT—laser interstitial thermal therapy; MEP—motor evoked potentials; mo—month; MRI—magnetic resonance imaging; MWA—microwave ablation; RFA—radiofrequency ablation; SEP—sensory evoked potentials; VB—vertebral body; yr—year.

## Data Availability

All data used in the present review are from previously published reports.
